# RNA G-quadruplex is resolved by repetitive and ATP-dependent mechanism of DHX36

**DOI:** 10.1038/s41467-019-09802-w

**Published:** 2019-04-23

**Authors:** Ramreddy Tippana, Michael C. Chen, Natalia A. Demeshkina, Adrian R. Ferré-D’Amaré, Sua Myong

**Affiliations:** 1 Department of Biophysics, Johns Hopkins University, Baltimore, MD 21218 USA; 2 Department of Chemistry, University of Cambridge, Cambridge, CB2 1TN UK; 3 Biochemistry and Biophysics Center, National Heart, Lung and Blood Institute, Bethesda, MD 20892 USA; 4 Physics Frontier Center (Center for Physics of Living Cells), University of Illinois, Urbana, IL 61801 USA

**Keywords:** Biochemistry, Single-molecule biophysics, Single-molecule biophysics

## Abstract

DHX36 is a DEAH-box helicase that resolves parallel G-quadruplex structures formed in DNA and RNA. The recent co-crystal structure of DHX36 bound G4-DNA revealed an intimate contact, but did not address the role of ATP hydrolysis in G4 resolving activity. Here, we demonstrate that unlike on G4-DNA, DHX36 displays ATP-independent unfolding of G4-RNA followed by ATP-dependent refolding, generating a highly asymmetric pattern of activity. Interestingly, DHX36 refolds G4-RNA in several steps, reflecting the discrete steps in forming the G4 structure. We show that the ATP-dependent activity of DHX36 arises from the RNA tail rather than the G4. Mutations that perturb G4 contact result in quick dissociation of the protein from RNA upon ATP hydrolysis, while mutations that interfere with binding the RNA tail induce dysregulated activity. We propose that the ATP-dependent activity of DHX36 may be useful for dynamically resolving various G4-RNA structures in cells.

## Abstract (web summary)

DHX36 is a G-quadruplex (G4) resolving helicase that targets both DNA-G4 and RNA-G4. Here the authors use single molecule FRET measurements and show that DHX36 resolves RNA-G4 structures by a mechanism involving an ATP-dependent, highly repetitive and stepwise refolding of RNA-G4 that differs from its DNA-G4 structures resolving mechanism.

## Introduction

DHX36 (also called RHAU and G4R1) is an RNA helicase that can resolve G-quadruplex (G4) DNA and G4-RNA with high specificity and affinity^1–6^. Accordingly, DHX36 is found enriched in G4 loci in cells^7^. DHX36 activity in targeting and unfolding G4-RNA is implicated in many mRNA and long non-coding RNA processes^8–11^. DHX36 helps promote translation of mRNAs with G4 motifs by unfolding the G4 structure^12^. In agreement, DHX36 knockout impedes translation of mRNAs containing G4 motifs at both 5′UTR and 3′UTRs^8,13^. In addition, DHX36 regulates the G4-harboring p53 mRNA after oxidative damage and localizes G4-forming mRNAs to stress granules, suggesting diverse, yet G4-specific functions of DHX36. Furthermore, the human telomeric G4-RNA is resolved by DHX36, contributing to the telomerase activity and telomere regulation.^7,14,15^ Aided by such G4 specific-helicases, cellular G4-RNA structures are efficiently resolved^16^. Thus, G4-RNA structures are likely undergoing dynamic folding and unfolding cycles^17,18^.

We and others have reported that DHX36 binds specifically to parallel conformation-G4-DNAs^5,19,20^ with a single-stranded (ss) tail^16^. The recent co-crystal structure of DHX36 bound to G4-DNA revealed that the resolved state entails a single guanine base pulled out of the folded G4 structure^21^, suggesting a partial rearrangement of G4 rather than a complete unfolding. Interestingly, we demonstrated that such activity by DHX36 is highly repetitive, monomer-driven and ATP-independent^20,21^. Structurally, the partial unfolding arises from rotation of the C-terminal domain and helicase core opening, resulting in pulling of one nucleotide out of the G4 registry. This is followed by subsequent refolding of G4. Cycles of such unfolding and refolding occur in succession, resulting in back-and-forth springing of the G4 structure between fully folded and partially disrupted states in an ATP-independent manner^21^.

Here, we report a distinctly different mechanism by which DHX36 acts on G4-RNA substrates. Unlike on G4-DNA, DHX36 binding to G4-RNA induces a stably unfolded state without repetitive pulling on the substrate. The unfolding is followed by successive cycles of gradual, ATP-dependent and stepwise refolding of G4-RNA. Such ATP-independent unfolding and ATP-dependent refolding of G4-RNA is repeated multiple times before the protein dissociates from the G4-RNA substrate. Mutational analysis reveals that disruption of G4 contacts induces rapid dissociation of DHX36 from G4-RNA whereas the changes in ssRNA contacting domains perturb the ATP-dependent stepwise refolding of G4-RNA, leading to an erratic and dysregulated motion of DHX36 on G4-RNA. Together, these studies describe a detailed mechanism for the G4-RNA resolving activity of DHX36.

## Results

### DHX36 displays ATP-dependent repetitive motion on G4-RNA

**Fig. 1:**
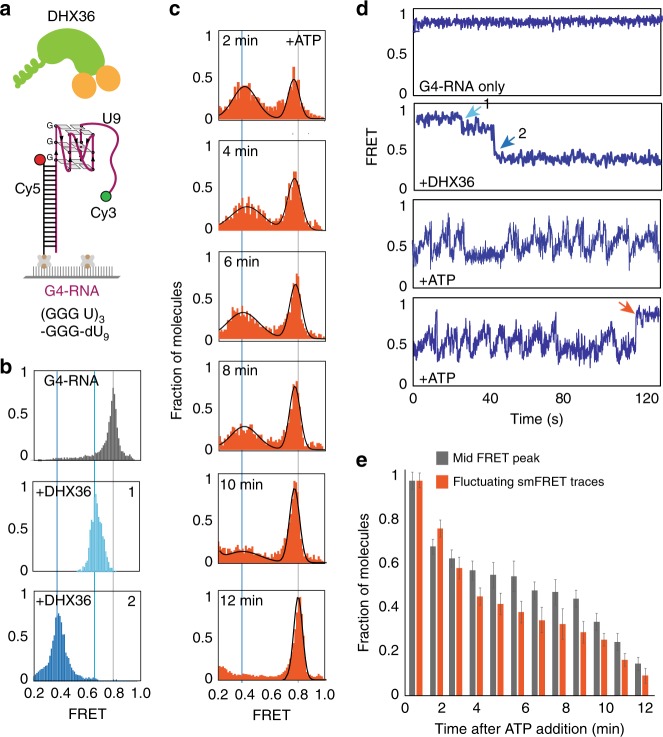
DHX36 displays repetitive cycles of unfolding and refolding of G4-RNA. **a** Single molecule FRET experimental scheme for testing DHX36 activity on G4-RNA. Two FRET dyes are attached to either end of G4. **b** FRET histograms taken at substrate only (top, 0.8 FRET peak), after DHX36 binds ssRNA (middle, 0.65 FRET peak) and after DHX36 engages with G4 (bottom, 0.4 FRET peak). **c** FRET histograms taken 0–12 min after 1 mM ATP is added. **d** Representative single molecule traces displayed in each condition. The bottom two traces represent continuous unfolding-refolding activity without (top) and with (bottom) DHX36 dissociation. **e** Quantitation of the fraction of middle FRET and FRET fluctuation over time plotted with standard error of means

In order to compare the activity of DHX36 on G4-DNA- vs. G4-RNA, we prepared two FRET constructs in which the G4 sequence consists of four runs of G triplets separated by single thymine and single uracil nucleotide in G4-DNA and G4-RNA, respectively (Table 1). The ss tails consisted of nine deoxy-thymine and nine uracil nucleotides in G4-DNA and G4-RNA, respectively (Supplementary Fig. 1A, Fig. 1a). Previously, we have shown that positioning the fluorophore at other than the 3′-end of the ssDNA prevents DHX36 from binding^20^, likely due to the tight contact between DHX36 and the substrate^21^. We have demonstrated that the high FRET state from this position of FRET pair dyes represents the G4 folded state. Protein binding to single-stranded tail results in high to mid FRET (0.65) transition whereas the unfolding of G4 by DHX36 or RecQ family helicases induce further FRET decrease to 0.4^19–23^. Based on this observation, we designed G4 constructs with an end-labeled short tail that allows DHX36 to bind and act on G4, and yields high FRET sensitivity^19,21^. As before, we employed a single molecule (sm) FRET assay to probe the DHX36 activity on both constructs.

**Table 1:** Sequences of RNA and DNA used in the study

Name	RNA and DNA sequences
18mer DNA with 5′-Cy5 and 3′ biotin	5′-/Cy5/-GCC TCG CTG CCG TCG CCA-biotin-3′ (down)
18mer RNA with 5′-Cy5 and 3′ biotin	5′-/Cy5/-rGrCrC rUrCrG rCrUrG rCrCrG rUrCrG rCrCrA-biotin-3′ (down)
RNA-GQ-U15	5′-rUrGrG rCrGrA rCrGrG rCrArG rCrGrA rGrGrC rUrUrG rGrGrU rGrGrG rUrGrG rGrUrG rGrGrU rUrUrU rUrUrU rUrUrU rUrUrU rUrU /3Cy3/-3′ (up)
RNA-GQ-U9	5′-rUrGrG rCrGrA rCrGrG rCrArG rCrGrA rGrGrC rUrUrG rGrGrU rGrGrG rUrGrG rGrUrG rGrGrU rUrUrU rUrUrU rUrU /3Cy3/-3′ (up)
RNA-GQ-A9	5′-rUrGrG rCrGrA rCrGrG rCrArG rCrGrA rGrGrC rUrUrG rGrGrU rGrGrG rUrGrG rGrUrG rGrGrA rArArA rArArA rArA /3Cy3/-3′ (up)
DNA-GQ-T9	5′-TGG CGA CGG CAG CGA GGC TTG GGT GGG TGG GTG GG TTT TTT TTT TTT TTT/3Cy3/-3′ (up)
RNA-GQ-UUA loop-U9	5′-rUrGrG rCrGrA rCrGrG rCrArG rCrGrA rGrGrC rUrUrG rGrGrU rArArG rGrGrU rArArG rGrGrU rArArG rGrGrU rUrUrU rUrUrU rUrU /3Cy3/-3′ (up)
DNA-GQ-U9 (RNA tail)	5′-UGG CGA CGG CAG CGA GGC UUG GGU GGG UGG GUG GG rU rUrUrU rUrUrU rUrU /3Cy3/-3′ (up)
RNA-GQ-T9 (DNA tail)	5′-rUrGrG rCrGrA rCrGrG rCrArG rCrGrA rGrGrC rUrUrG rGrGrU rGrGrG rUrGrG rGrUrG rGrGrT TTT TTT TT /3Cy3/-3′ (up)
Complementary strand for Cis annealing	5′-CCC A CCC A CCC A CCC AA G/iCy5/CC TCG CTG CCGTCG CCA /3Bio/-3

The G4-DNA alone yields high FRET (0.9) as depicted by a sharp high FRET peak in the FRET histogram (Supplementary Fig. 1B). The high FRET is due to the tight folding of parallel G4^19,20,24^ and a short tail of nine nucleotides, bringing the two dyes into close proximity. We have shown previously that the non-G4 forming sequence or G4 forming sequences with chemically modified guanines yield significantly lower FRET, further confirming the high FRET arising from G4 folding^20,21,23,25–28^. The representative smFRET trace displays a steady high FRET signal, consistent with the sharp FRET histogram peak. Upon addition of 5 nM DHX36, the high FRET peak immediately shifts to a broad mid FRET (0.5–0.6) (Fig. 1c, middle) with the smFRET trace showing a rapid fluctuation within this FRET range (Supplementary Fig. 1C). We interpret this fluctuation as arising from DHX36 partially and repetitively unfolding the G4 structure, generating successive cycles of unfolding and refolding of G4^20^. Our recent structure revealed that DHX36 disrupts G4 folding by pulling out a single nucleotide^21^. This is independent of ATP as similar fluctuations persist in the absence or presence of ATP (Supplementary Fig. 1C), consistent with our previous finding^20,21^.

G4-RNA exhibits high FRET (0.8) by itself (Fig. 1b, gray). Upon addition of DHX36, the FRET shifted first to 0.65 (Fig. 1b, light blue), followed by transition to 0.4, slightly lower than that of the G4-DNA bound by DHX36 (Fig. 1b, cyan). In contrast to the rapid FRET fluctuations seen with G4-DNA (Supplementary Fig. 1C), G4-RNA displayed a steady low FRET with no fluctuation when bound by DHX36 (Fig. 1d, second panel). The two steps of FRET decrease represent the initial contact of ssRNA tail (light blue arrow, labeled 1) followed by disruption of G4 (blue arrow, labeled 2), respectively (Fig. 1d). The two-step binding resembles that seen with G4-DNA^19^. The steady low FRET level reveals that G4 is stably engaged with DHX36, and partially disrupted by the protein. The measurements were taken after excess protein was removed by flowing buffer into the imaging chamber, confirming the tight binding of DHX36 on G4-RNA. When ATP was added, the FRET peak gradually shifted from 0.4 back to the 0.8 in 12 min, indicating that DHX36 slowly dissociated from G4-RNA (Fig. 1c). The individual smFRET traces taken at 2–10 min after the addition of ATP revealed FRET fluctuations indicative of DHX36 unfolding and refolding the G4-RNA in a repetitive manner, before dissociating from it (Fig. 1d, bottom, orange arrow). Such FRET fluctuations only occur in the presence of ATP and are significantly slower than the rapid ones seen with G4-DNA (Supplementary Fig. 1C). To test if the broad 0.4 FRET peak corresponds to fluctuating molecules, we quantified the fraction of middle FRET peak (0.4) obtained from the FRET histograms (Fig. 1e, gray bars) and the fraction of smFRET traces that display FRET fluctuations (Fig. 1e, orange bars). The similar pattern between the two fractions demonstrate that the 0.4 FRET peak arises primarily from the molecules that exhibit FRET fluctuations. As shown, FRET fluctuations occur for up to 10 min before DHX36 disengages from G4-RNA. Taken together, DHX36 displays an ATP-dependent, repetitive unfolding-refolding of G4-RNA.

### DHX36 monomer exhibits asymmetric motion

**Fig. 2:**
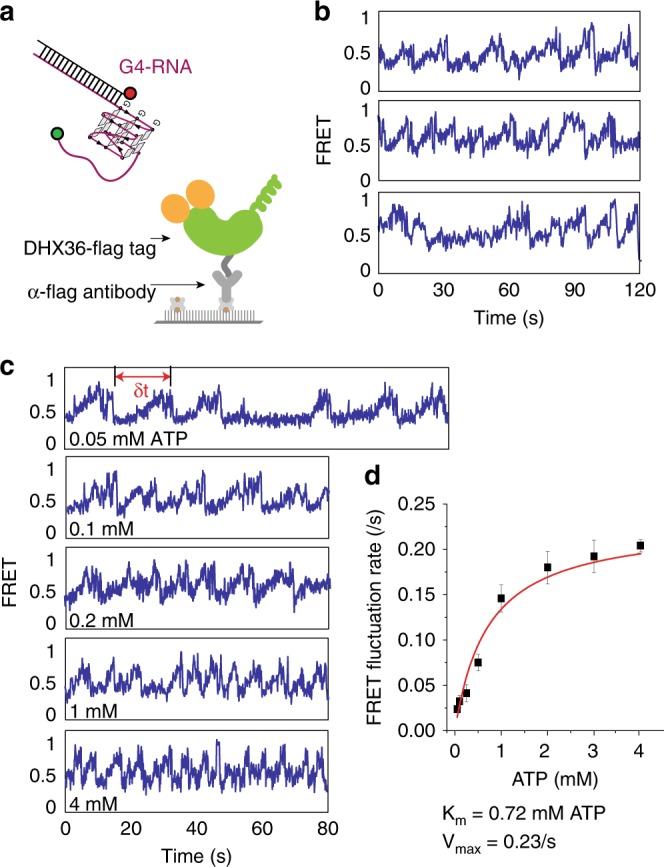
The repetitive DHX36 activity is monomer-driven and ATP dependent. **a** Experimental scheme for the reciprocal assay in which single molecules of DHX36 were immobilized to surface and FRET labeled G4-RNA was added. **b** Representative smFRET traces. **c** smFRET traces taken at varying concentrations of ATP from lowest (top) to the highest (bottom). **d** The rate of FRET fluctuation fitted to Michaelis-Menten kinetics, yielding *K*_m_ of 0.72 mM ATP and *V*_max_ of 0.23/s. The standard error bars were generated from over 300 FRET fluctuation events collected from over 75 single molecule traces taken from three sets of independent measurements

We have shown previously that the repetitive DHX36 activity on G4-DNA arises from a monomeric protein^20,21^. Based on our experimental scheme in which the measurement is taken after washing out excess protein by buffer flow (Fig. 1c), the repetitive activity of DHX36 on G4-RNA is likely due to a monomer of DHX36 rather than successive binding of multimers. To test this further, we immobilized individual DHX36 helicases by flag-antiflag interaction in sm surface (Fig. 2a)^29,30^. When the same G4-RNA FRET construct without biotin was added with ATP, we observed the same repetitive FRET fluctuation (Fig. 2b) as before (Fig. 1c), strongly suggesting that the repetitive activity of DHX36 is due to a monomer rather than successive binding of many molecules.

Next, we measured the DHX36 activity under varying ATP concentrations to examine the ATP dependence. At low ATP concentrations, the FRET fluctuation drastically slowed down (Fig. 2c, top). One prominent feature of the smFRET traces is an asymmetric pattern consisting of a gradual FRET increase followed by a rapid FRET decrease (Fig. 2b, c). Interestingly, only the gradual rise, but not the sudden decrease in FRET slows down as a function of ATP concentration, suggesting that the rapid drop in FRET is not ATP dependent. This is reminiscent of DNA translocases, as we reported previously^30–32^. Therefore, we interpret this pattern as arising from an ATP-dependent gradual refolding followed by an ATP-independent instantaneous unfolding of G4-RNA. The ATP-independent unfolding is consistent with the observation that DHX36 binding is sufficient to induce unfolding in the absence of ATP (Fig. 1b, c). Furthermore, we show that such activity of DHX36, which leads to eventual dissociation from G4-RNA, only occurs in ATP hydrolyzing conditions, i.e., both hydrolysable ATP and magnesium(II) are required (Supplementary Fig. 2). We also show that similar ATP-dependent repetitive activity of DHX36 was observed on substrates with longer polyuracil tail (U15), with a polyadenine tail (A9), or on a longer looped (UUA) substrate (Supplementary Fig. 3), indicating the conserved mechanism of DHX36 activity regardless of tail length, sequence and G4 composition.

### ATP-dependent G4-RNA refolding involves discrete steps

**Fig. 3:**
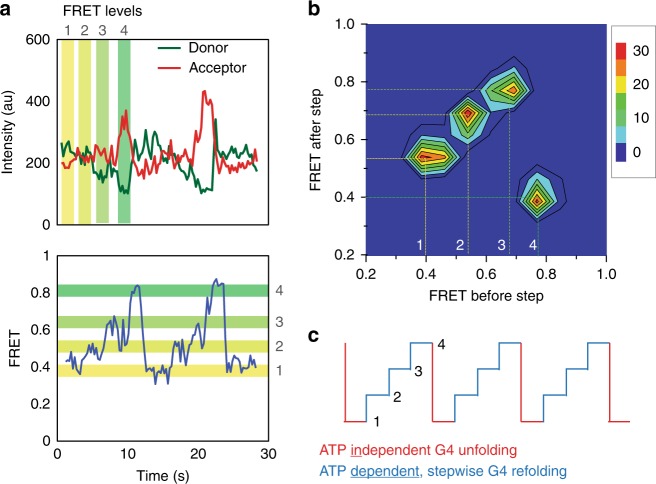
G4-RNA refolding entails discrete steps of DHX36 movement. **a** smFRET trace that shows anticorrelated change of donor (Cy3, green) and acceptor (Cy5, red) (top). FRET traces calculated from donor and acceptor intensity (bottom). **b** Transition density plot generated by taking FRET values before and after FRET transition for the successive FRET steps. **c** Schematic representation of FRET steps. One step FRET decrease corresponds to ATP independent G4 unfolding (red) while stepwise FRET increase indicates ATP dependent multistep refolding of G4

A closer examination of the smFRET traces revealed that FRET increases in several steps whereas the FRET decreases in one step. The representative FRET trace displays the intensity of Cy3 (green) and Cy5 (red) changing in a stepwise and anti-correlated manner (Fig. 3a, top) and calculated FRET increasing in discrete steps, followed by a rapid FRET decrease (Fig. 3a, bottom). We collected over 300 FRET values of each FRET steps from sm traces and plotted them into a transition density plot (TDP) (Fig. 3b). The TDP represents how FRET values change before and after individual steps, i.e., the first, second and third steps encompass FRET transitions from 0.4 to 0.52, 0.52 to 0.68, 0.68 to 0.78, respectively, followed by one-step decrease from 0.78 to 0.4, corresponding to the instantaneous FRET decrease. Taken together, the DHX36 activity can be summarized as an ATP-independent unfolding of G4-RNA (Fig. 3c, red line) followed by ATP dependent stepwise refolding of G4-RNA (Fig. 3c, blue line).

### ssRNA tail but not G4 induces ATP-dependent motion

**Fig. 4:**
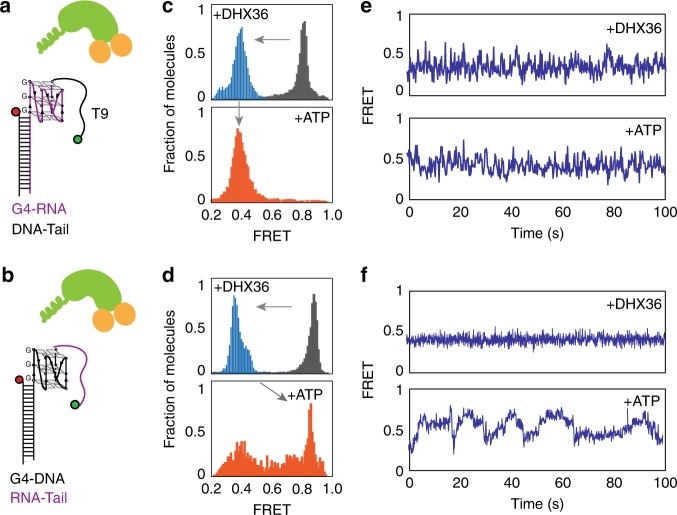
ATP dependent G4-RNA refolding arises from RNA tail, not G4. **a**, **b** Chimeric constructs of G4-RNA with ssDNA tail (A) and G4-DNA with ssRNA tail to which DHX36 was added. **c**, **d** FRET histograms for substrate only (gray), after DHX36 addition and buffer wash (blue) and after 1 mM ATP addition (orange). **e**, **f** Representative smFRET traces obtained in each condition as indicated

We asked what moiety of the G4-RNA construct is responsible for the ATP-dependent asymmetric motion of DHX36. Previously, we have shown that DHX36 binding and activity requires both the parallel G4 and the ss tail of DNA^19,20^. To test whether the G4 or the ssRNA tail is responsible for the ATP-dependent activity of DHX36, we prepared two RNA-DNA chimeras comprised of G4-RNA with ssDNA tail (Fig. 4a), or G4-DNA with ssRNA tail (Fig. 4b, Table 1). When DHX36 was added to the high FRET G4-RNA-ssDNA chimera, the FRET value shifted from 0.8 to 0.4, indicating DHX36 binding. Unlike the case of G4-RNA-ssRNA, the smFRET traces displayed rapid FRET fluctuations (Fig. 4c, e top) resembling the activity of DHX36 on G4-DNA-ssDNA tail (Fig. 1c, e middle). Addition of ATP did not change the FRET peak or the smFRET traces, i.e., FRET peak stayed at 0.4 for over 12 min and the individual traces exhibited a similar FRET fluctuations (Fig. 4c, e bottom). This clearly indicates that the G4-RNA alone is not sufficient to yield the ATP -dependent activity of DHX36. In addition, the ssDNA tail is sufficient to recapitulate the DHX36 activity observed for G4-DNA-ssDNA.

The DHX36 activity on the other chimera, G4-DNA-ssRNA tail, exhibited an activity similar to G4-RNA-ssRNA case. The G4-DNA-ssRNA exhibited a high FRET as before and DHX36 binding induced FRET change from 0.85 to 0.4, similar to all other cases (Fig. 4d, top). Individual smFRET traces, however displayed a static signal with no FRET fluctuations, indicating a stable interaction between DHX36 and the G4-DNA-ssRNA (Fig. 4f, top), resembling the case of G4-RNA-ssRNA tail (Fig. 1f, middle). In the presence of ATP, DHX36 induced an activity reminiscent of its activity on G4-RNA-ssRNA tail (Fig. 4f bottom). First, the DHX36 molecules dissociated from the construct in ATP dependent manner (in 12 min), evident from the FRET shifting back to 0.85 FRET (Fig. 4d, bottom). Second, FRET fluctuation was induced only in the presence of ATP. Third, the FRET displays a slow, gradual and periodic manner (Fig. 4f, bottom) although the fluctuation pattern became less asymmetric (Supplementary Fig. 4). Taken together, we show that the ssRNA tail is responsible for generating the ATP-dependent G4 refolding, suggesting a dominant role played by the OB fold and RecA-like domains of DHX36 that interact primarily with the ssDNA or ssRNA tail^21^.

### Mutational analysis of DHX36 activity

**Fig. 5:**
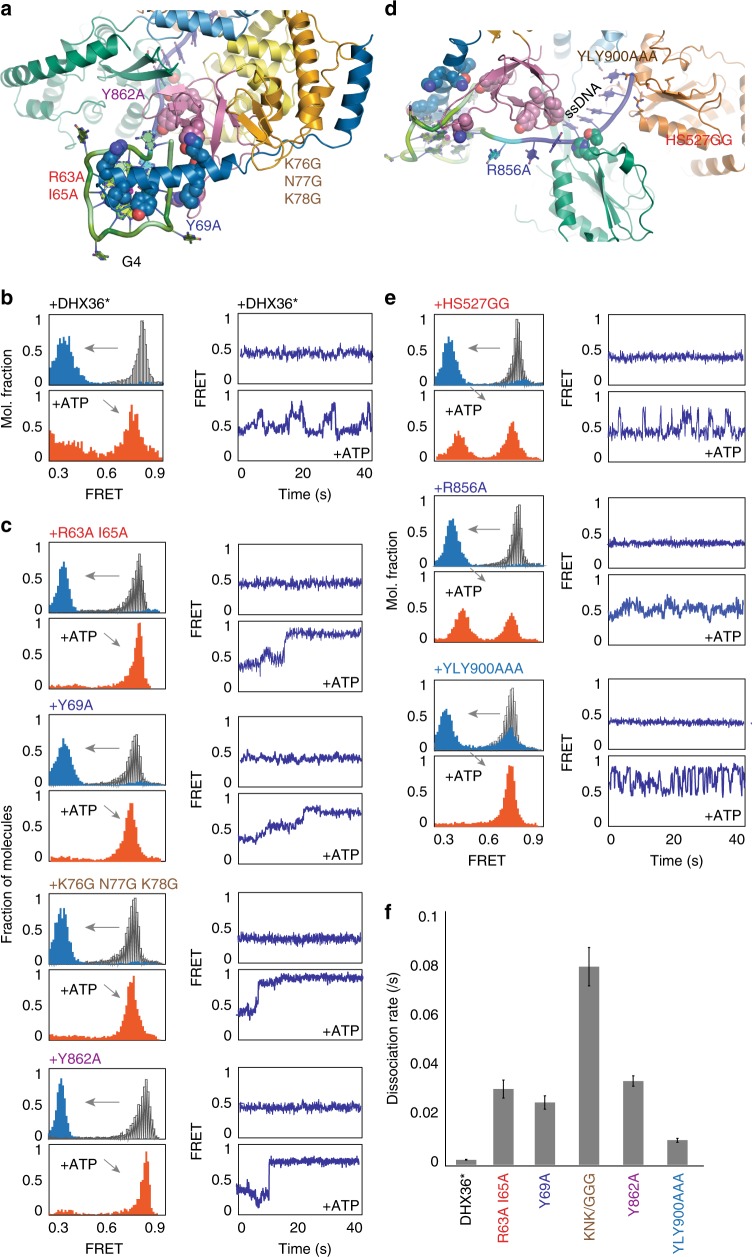
Point mutations reveal a clear partition in DHX36 function. **a** Site-directed mutations introduced to DHX36 G4 acting elements. **b** Histogram and smFRET traces obtained for DHX36*, a bovine version used in previous structural study. **c** Histograms and smFRET traces generated for all four mutants. They all bind G4-RNA stably, but exhibited fast dissociation upon ATP hydrolysis. **d** Biochemical mutations introduced to ssDNA interaction domain of DHX36. **e** Histogram and smFRET traces obtained for HS527GG (top), R856A (middle) and YLY900AAA (bottom). **f** Dissociation rate of DHX36 mutants upon ATP hydrolysis. The standard error bars were generated from over 200 molecules collected from three sets of experiments for each mutant

**Fig. 6:**
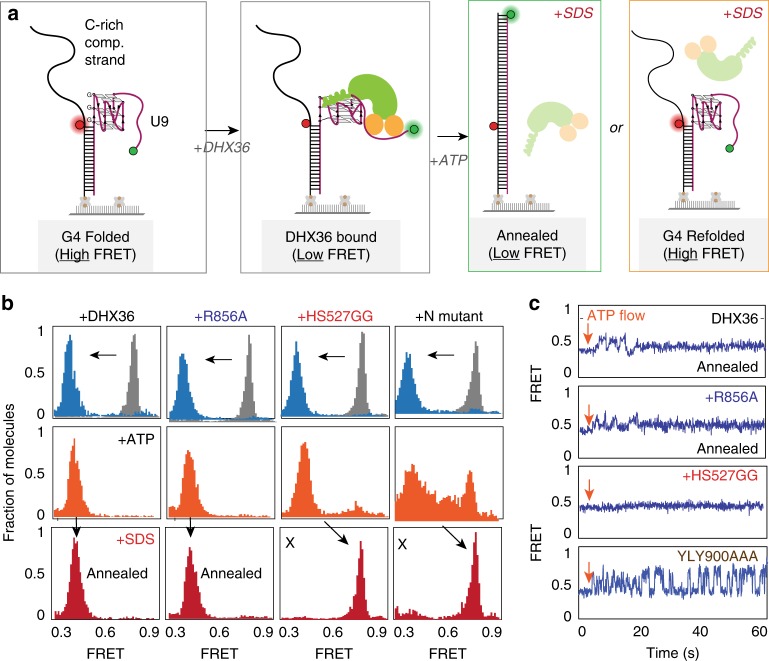
Annealing of G4 to complementary cis-strand depends on successful unfolding of G4. **a** Schematic of *cis*-annealing assay. ATP-dependent G4 resolving activity of DHX36 may result in annealing or refolding of G4. **b** FRET histograms taken at RNA-only (gray), DHX36-bound (blue), ATP added (orange) and SDS applied (dark red) for DHX36 and three mutants. **c** smFRET traces taken for each construct

We took advantage of site-directed mutants of DHX36 generated to perturb interaction with either the G4 or the ssDNA (Figs. 5a, 6a)^21^. First, we tested if the bovine DHX36 used for structural analysis recapitulates the activity of human DHX36 used in this study. Both the FRET shift and individual smFRET traces revealed that the same activity is exhibited by the bovine DHX36 (Fig. 5b). The first group of mutants, R63A/I65A, Y69A, K76G/N77G/K78G, and Y862A are located in regions that contact the G4 structure including the important DSM moiety which caps the top of flat tetrad surface, conferring the parallel G4 binding specificity (Fig. 5a)^5,21^. In all four mutations, the protein binds and unfolds G4-RNA in the absence of ATP, i.e., FRET shifts to 0.4. Again, the FRET histograms are taken after buffer wash, therefore protein binding to G4-RNA is stable in all cases. We have demonstrated previously that DHX36 first binds single stranded tail, which brings high FRET (0.8) to a mid FRET (0.6) and subsequent partial unfolding of G4 induces further FRET decrease to 0.4 state for all DSM and OB subdomain mutants^21^. Consistently, the 0.4 FRET value shown here represents the state in which DHX36 is engaged stably with both ssRNA and G4. In the presence of ATP, however, they rapidly dissociate from G4-RNA without exhibiting the ATP-dependent asymmetric motion (Fig. 5c). This suggests that the incomplete grip of G4 induces protein to dissociate when induced by ATP hydrolysis. The dissociation rate of individual mutants were calculated based on dwell time analysis i.e collecting the time interval between the flow of ATP and the moment of protein dissociation, i.e., rapid FRET increase to 0.8 (Fig. 5f).

The second group of mutants, HS527GG (in the RecA2 subdomain), R856A and YLY900AAA (in the OB subdomain), interact directly with the ss nucleic acid tail (Fig. 5d)^21^. All three mutants bind and unfold G4-RNA although the binding affinity is slightly diminished in YLY900AAA case (Fig. 5e, f left panel). The addition of ATP leads to partial dissociation of DHX36 albeit less than the wild type, suggesting a diminished propensity to dislodge from the G4 substrates. Moreover, DHX36 induces FRET fluctuations of irregular pattern, i.e., HS527GG displays abrupt peaks to high FRET rather than gradual FRET increase while R856A generates FRET fluctuations in a reduced FRET range (0.4–0.6) and the YLY900AAA induces rapid FRET fluctuations which appears to be dysregulated or frustrated activity (Fig. 5e). In summary, the G4 contacting residues are pivotal for stable grip or anchoring, while those interacting with ssRNA are crucial for ATP-dependent folding-unfolding activity, likely coordinated by the OB folds and RecA-like domains which translocate on ssRNA fueled by ATP hydrolysis.

### *Cis*-annealing depends on ATP-dependent G4 resolution

Next, we tested if the ATP-dependent G4 resolving activity can induce *cis*-annealing^20^. We hypothesized that the annealing between G4 and complementary strand positioned in the same molecule can only occur if DHX36 successfully resolves the G4 and thereby exposes the G4 strand. If the resolving activity is insufficient, G4 structure will refold (Fig. 6a). We tested the wild type DHX36 and all the site-directed mutants tested above (Fig. 5). The annealing RNA construct exhibits a similar high FRET by itself (Fig. 6b, left top, gray). Binding of DHX36 resulted in an immediate FRET shift to a low value (~0.35) (Fig. 6b left top, blue). Addition of ATP did not change the overall FRET value without shifting back to the high FRET state, suggesting that the steady low FRET represents the annealed state of RNA. We confirmed that this low FRET represents fully annealed state (Supplementary Fig. 5). However, since the low FRET may arise from the annealed state (Fig. 6a, third schematic) or DHX36 bound to G4 without successful annealing (Fig. 6a, second schematic), we applied 0.1% SDS to denature and dislodge DHX36 from substrate. This led to a stable low FRET, strongly suggesting that the G4 strand is stably annealed to C-rich complementary strand (Fig. 6b, left bottom). The same experiment performed in the absence of ATP resulted in returning back to high FRET which reflects refolding of G4 (Supplementary Fig. 5A), further suggesting that the low FRET state seen after SDS treatment represents annealed state. We observed a similar signature of low FRET transition in all mutants (Supplementary Fig. 5) except for the two mutants, HS527GG and YLY900AAA, both of which interact intimately with ss nucleic acid which exhibited irregular resolving activity in the presence of ATP (Figs. 5e, f and 6b). Both mutants induced high FRET upon SDS addition, signifying that the ATPase deficient mutants did not lead to G4 unwinding (Fig. 6b, right two columns). Furthermore, the smFRET traces of DHX36 and R856A (OB fold mutant) both displayed that upon ATP flow, FRET fluctuation occurs before reaching into a stably annealed state. In contrast, the HS627GG displayed no signal change whereas the YLY900AAA exhibited rapid FRET fluctuation without reaching the annealed state (Fig. 6c). The traces collected at the time of SDS flow displays a rapid FRET change from low to high, suggesting that the protein dissociation led to refolding of G4 (Supplementary Fig. 5).

## Discussion

DHX36 is unique in its ability to resolve both G4-DNA^1^ and G4-RNA^2^. Potential G4 forming sequences in human genome are abundant (>350,000), yet unevenly distributed. The enrichment of G4 forming sequences in promoters of oncogenes and regulatory genes strongly suggests their potential role in regulating transcription of important genes^33^. The presence of G4 in the human genome was confirmed by antibody probing and sequencing^34,35^ and more recently by BioTASQ^18^. Telomeric G4 is another site of G4 overrepresentation^36^. Therefore, both the promoter G4 and telomeric G4 are targeted for anticancer therapeutic by small molecules^37^. The recent co-crystal structure of DHX36-G4-DNA clearly demonstrated the nature of intimate contact between the protein and a parallel G4 structure^21^. DHX36 binding requires two parts; parallel G4 and a ss tail of at least 8–9 nucleotides^19,20^. Our previous and current findings indicate that upon engaging with the G4-DNA, DHX36 repetitively pulls out one guanine and releases for G4 to resume folding, resulting in a tug of war-like motion. This partial unfolding and refolding cycle is ATP-independent and continues for many cycles without dissociation from the substrate (over 20 min). Nevertheless, the motion discontinues when a C4 complementary strand anneals with the G4 strand^20^, suggesting that the partial unfolding induced by DHX36 is sufficient to disrupt the G4 structure to allow for annealing with the complementary strand. Such activity can lead to restoring of the duplex DNA configuration, which can contribute to genome integrity.

Unlike the G4-DNA which forms in duplex DNA, G4-RNA forms in single stranded context such as mRNA and long noncoding RNA. In particular, the G4-RNA forming sequences are highly enriched in 5′UTR, 3′UTR and the 5′ side of the first intron of numerous genes^38,39^. Therefore, stable folding of G4-RNA can potentially modulate translational activity unless resolved by helicases such as DHX36. Indeed, DHX36 deletion led to heart defect and embryonic lethality in mice due to G4-RNA formed in 5′UTR of Nkx2-5 mRNA which requires DHX36 activity for translation^13^. Here, we present a mechanism by which DHX36 resolves G4-RNA in an ATP-dependent and repetitive manner. The mechanism is unique in several aspects. First, the binding of DHX36 which does not depend on ATP, leads to unfolding of G4-RNA and the binding is stable against buffer wash. This reflects an inherent binding mode which is built to twist open the G4 structure. Second, ATP hydrolysis leads to slow, gradual, and stepwise refolding of G4-RNA (FRET recovers to the level of folded G4-RNA). This may represent ATP binding in a closed core conformation (FRET 0.5), hydrolysis of ATP to ADP (FRET 0.7), ADP release (FRET 0.8) followed by phosphate release which springs back to unfolded G4 (FRET 0.4), yet our experiment cannot directly probe such motion. Determination of such detailed mechanism is warranted for future study. Third, the asymmetric ATP independent unfolding followed by ATP dependent refolding of G4-RNA is driven by a monomer DHX36 and repeated many times in succession. Fourth, such repeated cycles of activity leads to dissociation of DHX36 from G4-RNA substrate. Fifth, the ATP-dependent refolding of G4-RNA arises from the RNA tail rather than the RNA-G4. If such activity occurs in cellular RNA, for example at a parallel G4 formed in 5′UTR, DHX36 monomer will maintain contact with the RNA-G4 while dynamically modulating the structure until it encounters a cognate molecule such as ribosomes that need to gain access the 5′ side of the mRNA. This unique mechanism can potentially serve to; (i) resolve G4 structure, (ii) prevent the reformation of G4, (iii) protect the G4 from degradation, (iv) shield G4 from other proteins, (v) keep the G4 in dynamic state, and (vi) release from G4 structure eventually.

**Fig. 7:**
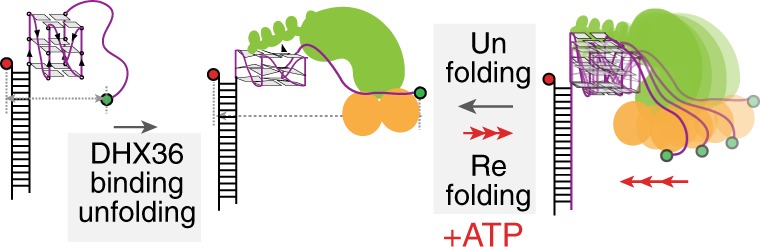
Schematic summary for DHX36 activity on G4-RNA. The ATP-independent unfolding of G4-RNA is followed by ATP-dependent and repetitive refolding which generates a highly asymmetric pattern of DHX36 activity

Our mutational analysis revealed functional roles for two DHX36 subdomains. First, all mutations in DSM resulted in rapid dissociation upon ATP hydrolysis, strongly suggesting the role of the DSM in anchoring DHX36 to G4 structure. Interestingly, the Y69A mutation in DSM caused DHX36 to disengage from DNA-G4 upon buffer wash^21^, yet the same mutant remained bound to RNA-G4 even after the buffer flow, indicating a different DSM-G4 interaction between DNA and RNA. Despite its fast dissociation upon ATP hydrolysis, the DSM mutations led to efficient annealing between the G4 and the complementary C-rich strand. For K76G N77G K78G mutant, the protein appears to detach from the substrate immediately after the flow of ATP (Fig. 5c), likely representing at most a single round of ATPase activity that dislodges the protein. Such may be plausible if the single round of proper ATPase activity is enough to unfold the G4 structure, leading to rapid pairing with the C-rich strand. In contrast, all mutations in the RecA2 and OB subdomains that alter ssRNA binding produced irregular motions when hydrolyzing ATP, indicating their role in translating ATP hydrolysis to coordinate the G4 refolding activity. The proteins did not dissociate in this case likely due to the intact DSM contact with the G4. Contrary to the DSM mutants, two OB subdomain mutants were insufficient to induce annealing with the complementary strand, suggesting that the irregular motions (Fig. 5e, f) did not properly unfold the G4. Taken together, our study reveals a mechanism of DHX36 which is built for specifically resolving parallel G4 structure which forms in the context of ssRNA (Fig. 7).

## Methods

### RNA substrate preparation

All RNA and DNA substrates are listed in Table 1 (synthesized and HPLC-purified by Integrated DNA Technology Inc). Substrates were designed with proper chemical modification including biotin for immobilization to NeutrAvidin surface and amine modification for NHS-ester dye labeling. For labeling of RNA, we mixed 25 μL of 200 μM of ssRNA containing amine modification with 0.2 mg of NHS ester-conjugated Cy3 or Cy5 dye in 100 mM NaHCO_3_ buffer, pH 8.5. The mixture was incubated overnight and the excess dye was removed by ethanol precipitation performed twice. The complementary ssRNAs or RNA-DNA hybrids were annealed by mixing each strand in an annealing buffer RNase free T50 (10 mM Tris, pH 8, and 50 mM NaCl), heating to 85 °C for 2 min, and gradually cooled at the rate of 2 °C/min until it reached 50 °C, followed by 5 °C/min decrease until it reached 4 °C on a thermocycler.

### DHX36 purification and RNA-DHX36 interaction conditions

Wild type and mutant DHX36 were purified by transforming RHAU encoding plasmid (codon optimized cDNA from GeneScript, Inc., NJ, USA) into BL21 (DE3) E. coli strain and inducing the protein expression by IPTG at 14^ o^C for overnight to reach OD of 1.2. The cell lysate was treated with 2× Sigma protease inhibitor mixture, 0.01 mM PMSA and 15 mg/ml leupeptin and applied to TALON cobalt bead by means of hexahistidine tag (Clontech). After washing three times with SSC (4×) with BME (0.5 mg/ml) containing buffer, the protein was eluted from the column by elution buffer containing 0.7 M histidine, pH 6.0, 8.6 mM BME and 1× Sigma protease inhibitor mixture. For second phase of elution, CMYC G4 DNA bound streptavidin paramagnetic beads were prepared as instructed (CGSPB, Thermo Scientific, USA). The eluant was applied to CGSPB and washed two time with cold SSC (4×) with 0.1% lactalbummin and 0.5 mg/ml BME. The elution was performed by adding ATP containing buffer. The purified enzyme stock was stored at −80  °C^20,21^. Single-molecule reactions were performed with 10 nM DHX36 in RNAse free buffer containing 50 mM Tris-HCl, pH 7.6, 50 mM KCl, 50 mM NaCl, 0.5 mM MgCl_2_, 10 percent glycerol and 1 mM ATP or other ATP analogs including ATPγS, AMP-PNP, ADP-AlF4. For ATP titration measurements ATP concentrations ranging from 10 μM to 4 mM ATP was used.

### Single-molecule FRET assays and data acquisition

Single-molecule FRET experiments were carried out on quartz slides (Finkenbeiner). To minimize nonspecific interaction of DNA, RNA or protein the quartz slides (Quartz Slides (1”x3”; 1 mm thick), For Prism type TIR. G. Finkenbeiner Inc.) and coverslips were passivated by treating with polyethylene glycol (PEG). Methanol, acetone, and potassium hydroxide were used to clean the slides and coverslips. Further, slides were burned, treated with amino silane, and coated with a mixture of 98% mPEG (m-PEG-5000; Laysan Bio) and 2.0% biotin PEG (biotin-PEG-5000; Laysan Bio)^40^. Partial duplex RNA or DNA molecules were annealed and immobilized on the PEG-passivated surface via biotin–NeutrAvidin interaction at the concentration range of 10–50 pM. Excess molecules were washed away with RNase free T50 buffer. An oxygen scavenging system [0.8 mg/mL glucose oxidase, 0.8% glucose, ∼5 mM Trolox (filtration to remove undissolved trolox), and 0.02 mg/mL catalase] was added to the imaging buffer for all smFRET measurements to minimize photobleaching of dyes. All experiments and measurements were carried out at room temperature (23 °C ± 1 °C). We used custom-built prism based total internal reflection fluorescence microscope for all single-molecule FRET assays. A solid-state 532 nm laser (Spectra-Physics) was used for smFRET measurement. Emission signal was separated by a dichroic mirror (cut off = 630 nm) and detected by an EMCCD camera (Andor). Data were recorded with a time resolution of 50–300 ms and analyzed with scripts written in interactive data language (IDL) to give fluorescence intensity time trajectories of individual molecules.

### smFRET data analysis

Matlab scripts have been used to analyze FRET efficiency, E, calculated as the intensity of the acceptor channel divided by the sum of the donor and acceptor intensities. To generate sm FRET histograms, we have collected data from over 6000 molecules and were fitted to Gaussian distributions using Origin software 8.0. For histogram analysis, we take short (3–5 s) movies from 20 different sm imaging surface with each movie yielding approximately 300–400 FRET values. We combine all 6000–8000 FRET values to build one histogram. The initial four frames taken from 300–700 ms duration are taken from each of ~6000 molecules are taken to build each smFRET histograms. Dwell times were analyzed by obtaining time intervals corresponding to each FRET state. TDP was created by Origin 8.0 software. The mean values with standard error of means were plotted.

### *Cis* annealing assay

Ten microliters of GQ-containing ssRNA with 3′Cy3 (Table 1) was heated up to 85 °C for 2 min by thermocycler in 10 mM Tris⋅HCl, pH 7.5, 150 mM KCl buffer. Temperature was gradually decreased at 2 °C/min until it reached 30 °C, followed by 5 °C/min until it reached 4 °C. Subsequently, 10 μL of 10 μM Cy5-labeled complementary strand was (Table 1) added to GQ-containing strand and incubated at room temperature for 5 min. The annealed mixture was immediately diluted to 100 pM in 10 mM Tris⋅HCl, pH 7.5, and 100 mM KCl for surface immobilization.

### Reporting summary

Further information on research design is available in the Nature Research Reporting Summary linked to this article.

## Supplementary information

Supplementary Information

Reporting Summary

Source Data

## Data Availability

The Source Data underlying Figs. 1e, 2d, 3b and 5 are provided as a Source Data file. Other data are available from the corresponding author upon reasonable request.
