# Supplementary material for: RNA G-quadruplex is resolved by repetitive and ATP-dependent mechanism of DHX36
Source: Nat Commun. 2019 Apr 23;10:1855. doi: 10.1038/s41467-019-09802-w (PMC6478676; doi:10.1038/s41467-019-09802-w)
Supplement: Supplementary file 1 — Supplementary Information [file 41467_2019_9802_MOESM1_ESM.pdf]

RNA G-quadruplex is resolved by repetitive and ATP-dependent mechanism of DHX36

Supplementary Information

Tippana et al,

Supplementary Figure 1. DHX36 rearranges G4-DNA structure repetitively. (A) The schematic of experiment in which FRET labeled G4-DNA substrate was immobilized to single molecule surface and DHX36 was added. (B) High FRET obtained for G4-DNA folded structure shifts to a broad mid FRET after addition of DHX36 and ATP. As previously demonstrated, the broad FRET peak result from highly repetitive, yet ATP-independent unfolding-refolding activity of DHX36[1, 2]. We note that the addition of ATP made FRET fluctuation slower, resulting in a different FRET distribution. Such difference, however was not seen when we tested various types of DNA-G4 construct in our previous work[1]. Therefore, this difference may be due to the particular G4 construct that we use in this study. (C) Representative smFRET shows a steady high FRET signal when G4-DNA is by itself. FRET fluctuates repetitively upon addition of DHX36, likely due to repetitive partial unfolding of G4-DNA. Such activity continues in the presence of ATP (1mM).

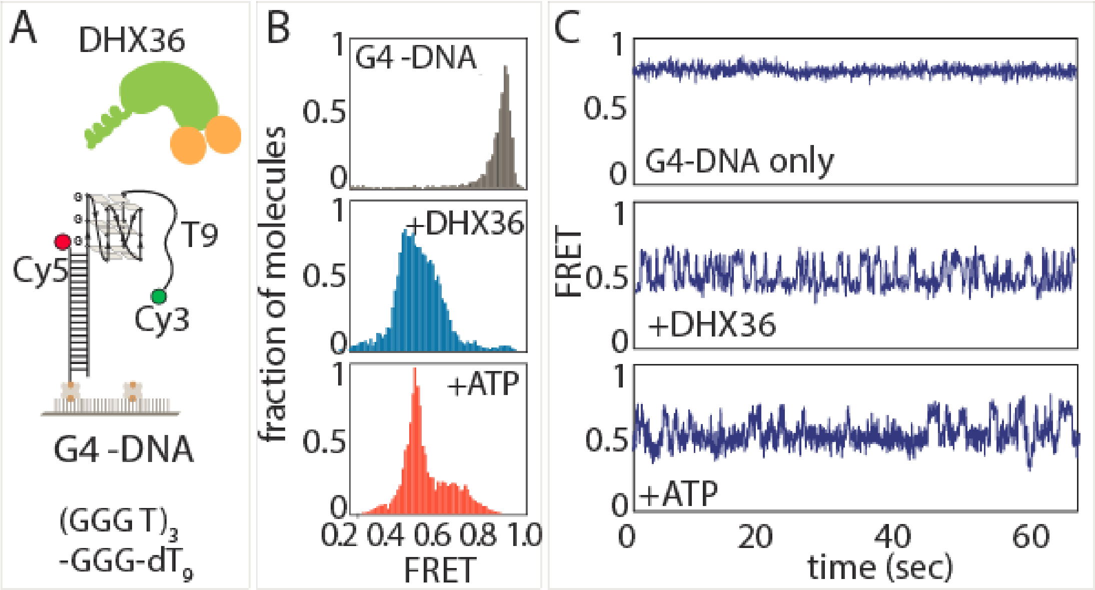

Supplementary Figure 2. DHX36 dissociation from RNA-GQ is ATP hydrolysis dependent. The FRET histogram shift from 0.4 to 0.8 which signifies dissociation of DHX36 from RNA-GQ occurs only in the presence of ATP hydrolyzing condition i.e in the presence of both ATP and magnesium (pink labels in A, B). Each histogram was taken approximately 10 minutes after each condition was applied. In addition, the rate of dissociation is correlated with the concentrations of magnesium (A) and ATP (B). (C) DHX36 stays bound to RNA without dissociating in the presence of non-hydrolyzable ATP analogs, AMP-PNP and ADP-Alf.

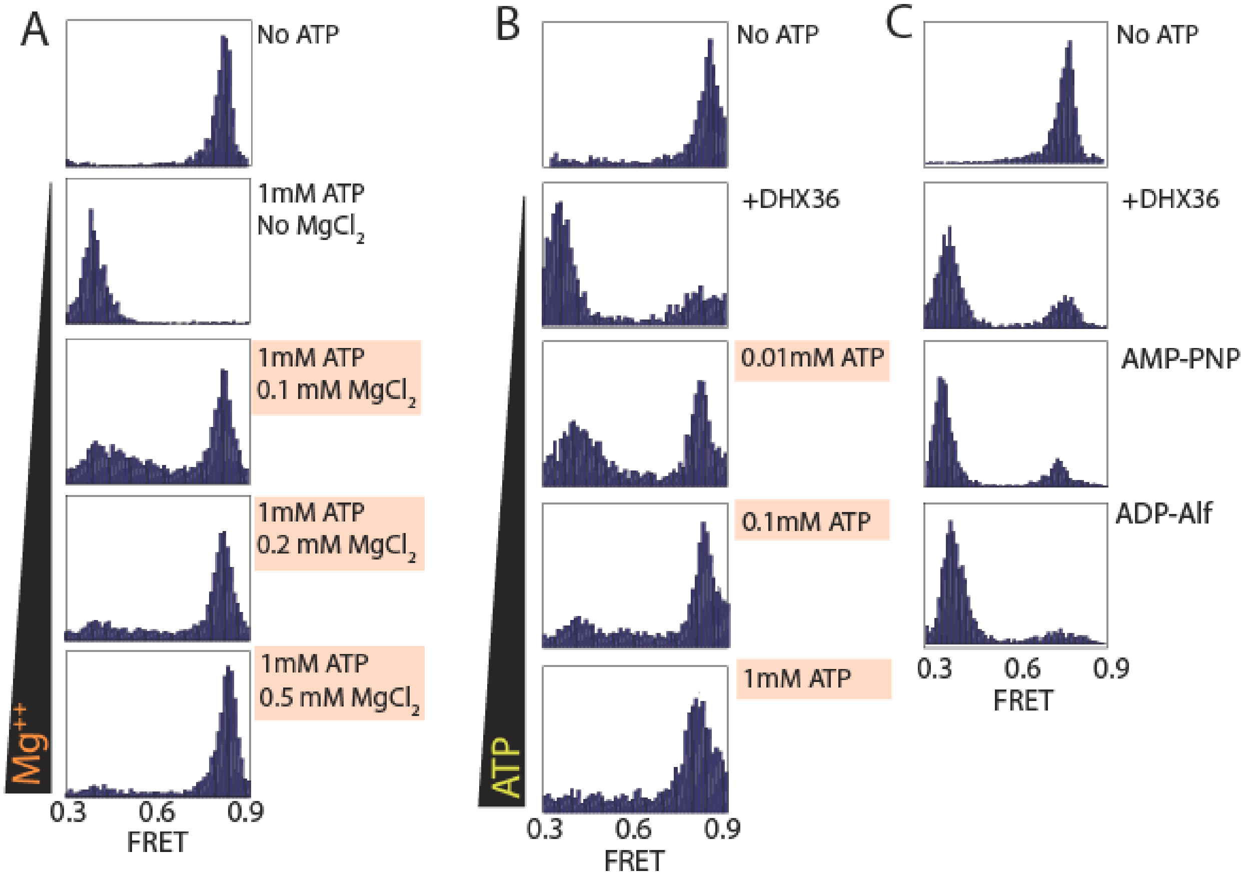

Supplementary Figure 3. Repetitive and asymmetric DHX36 activity persists regardless of tail composition, length and loop length. DHX36 activity was tested on three different RNA-GQ constructs. First, the tail of U9 was replaced by A9 (poly Adenine, left top). Second, the tail was lengthened to U15 (right top). Third, the loop sequence of single U was replaced by UUA in all three loops (bottom, left). In all cases, we observed similar FRET histogram change and ATP dependent repetitive FRET fluctuation followed by subsequent dissociation. The dissociation was delayed on U15, likely due to the long tail stabilizing DHX36 binding.

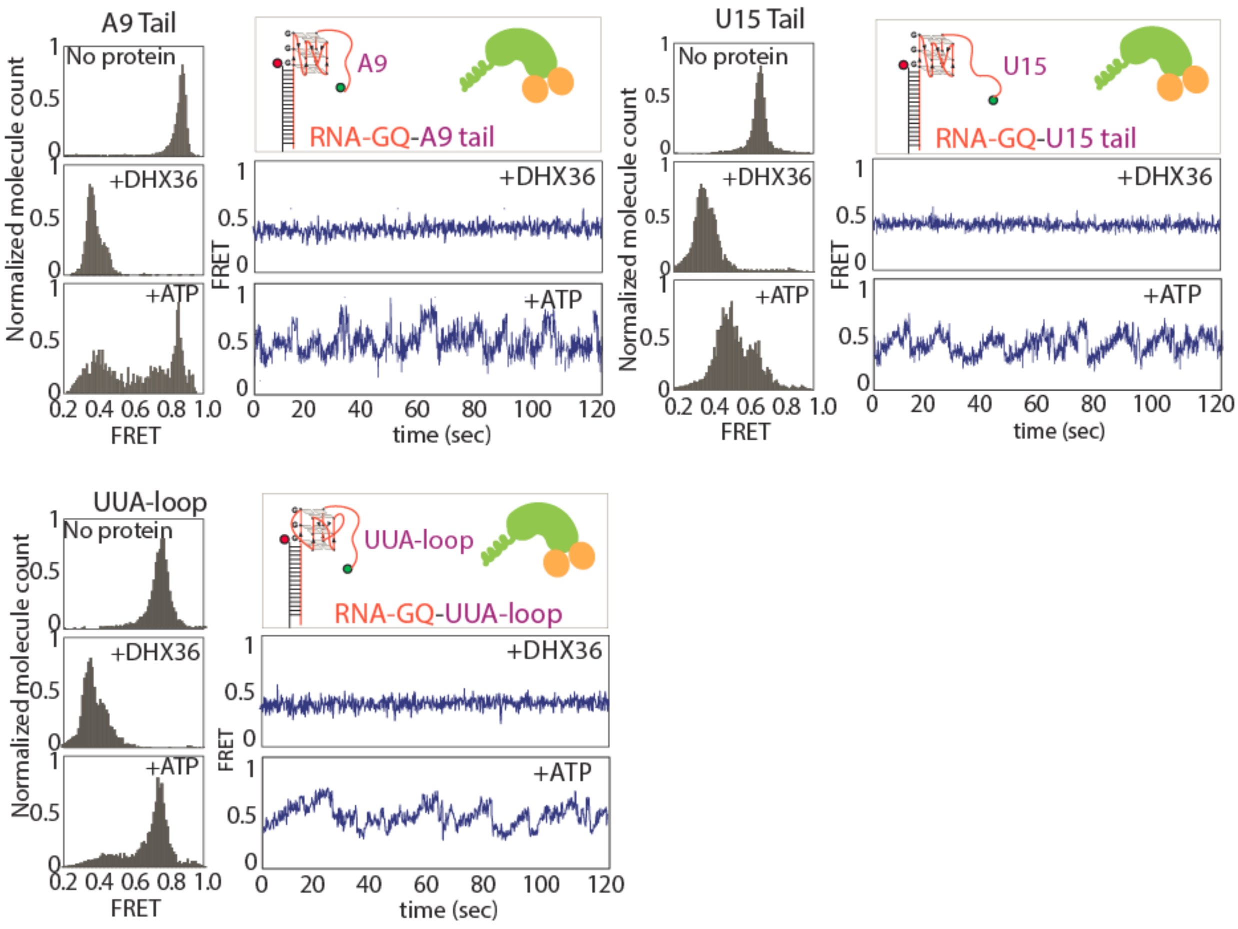

Supplementary Figure 4. DHX36 activity on DNA-GQ with RNA tail shows less asymmetric pattern. DHX36 added to DNA-GQ with RNA tail produced FRET traces that were less asymmetric than the ones on RNA-GQ with RNA tail (Fig. 1, 2).

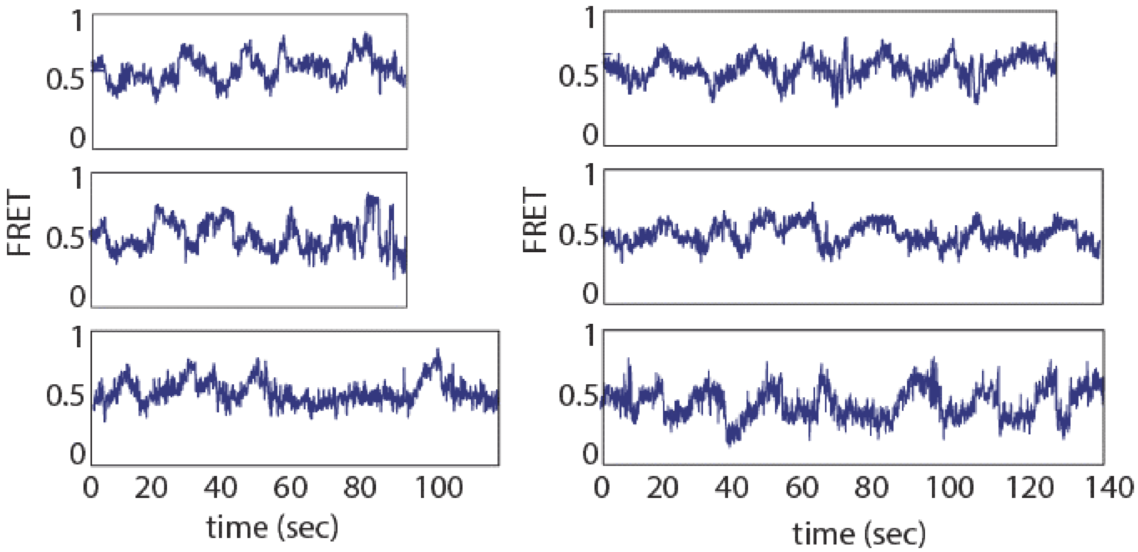

Supplementary Figure 5. Cis-Annealing of DHX36 mutants and SDS flow. (A) FRET histogram of RNA alone (gray), protein added (blue), ATP added (orange) and SDS flowed in (dark red). DHX36 binding alone does not lead to annealing, but all GQ contacting mutants of DHX36 lead to annealing between G4 and C-rich complementary strand upon ATP addition. Addition of SDS (to remove the protein from RNA) confirms annealing. (B) Flowing in ATP induces FRET fluctuation (partial unfolding of G4) which results in a stable low FRET signal signifying the stably annealed state. (C) Flow of SDS does not induce any further FRET change in wildtype DHX36, R856A, R63A/R65A, Y69A, Y862A, K76G/N77G/K78G. In contrast, FRET rapidly shifts to high value in OB-fold mutations, HS527GG and YLY900AAA, indicating G4 refolding which resulted from lack of G4 unfolding.

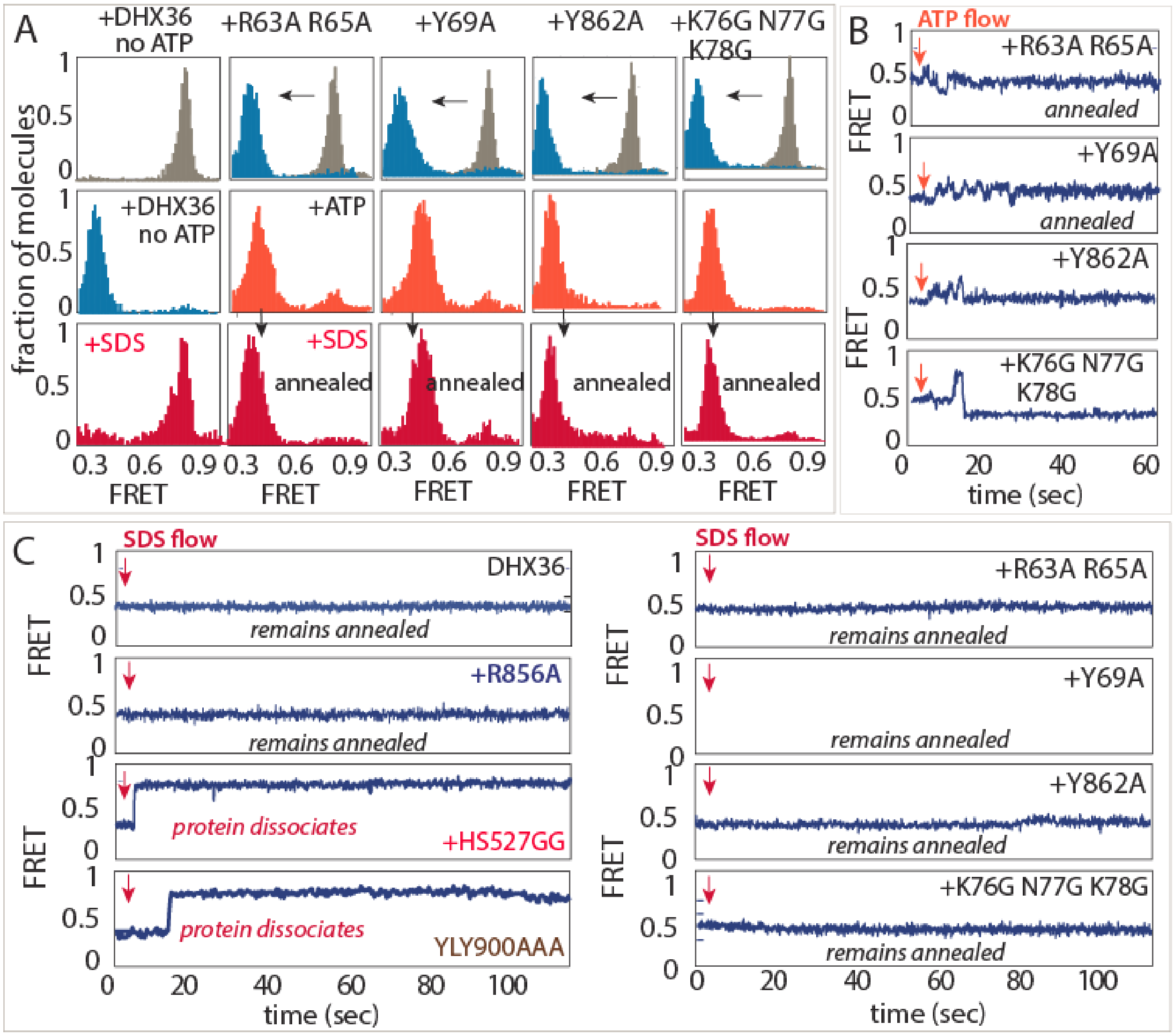

Supplementary References

1. Tippana R, Hwang H, Opresko PL, Bohr VA, Myong S. Single-molecule imaging reveals a common mechanism shared by G-quadruplex-resolving helicases. *Proc Natl Acad Sci U S A* **113**, 8448-8453 (2016).

2. Voter AF, Qiu Y, Tippana R, Myong S, Keck JL. A guanine-flipping and sequestration mechanism for G-quadruplex unwinding by RecQ helicases. *Nature communications* **9**, 4201 (2018).
